# Supplementary figures and images for: Identification of candidate genes associating with soybean cyst nematode in soybean (Glycine max L.) using BSA-seq
Source: PeerJ. 2024 Oct 21;12:e18252. doi: 10.7717/peerj.18252 (PMC11505975; doi:10.7717/peerj.18252)

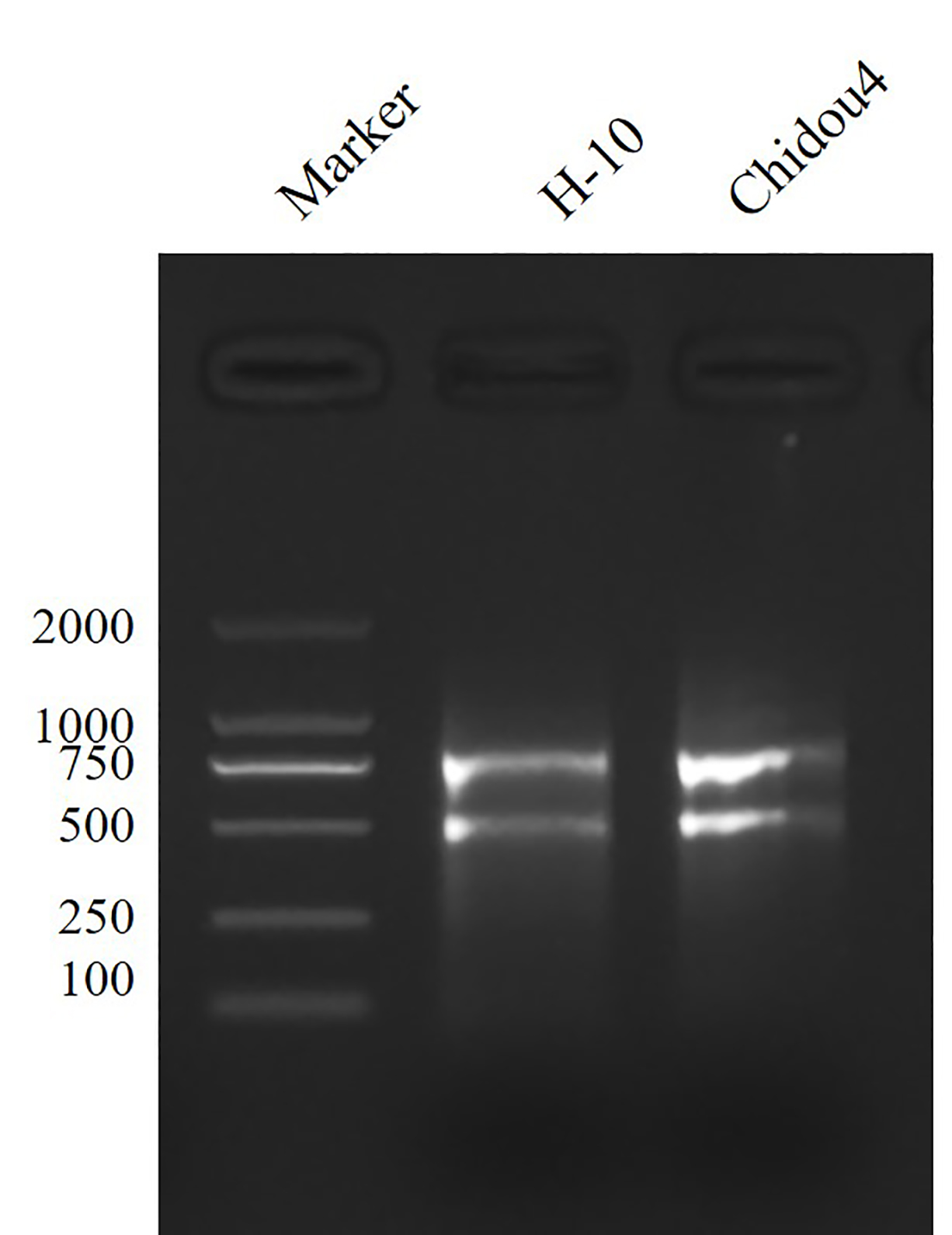

Supplement: Figure S1 — Marker, for DL2000 DNA Marker (100 bp–2000 bp). [file peerj-12-18252-s001.jpg]

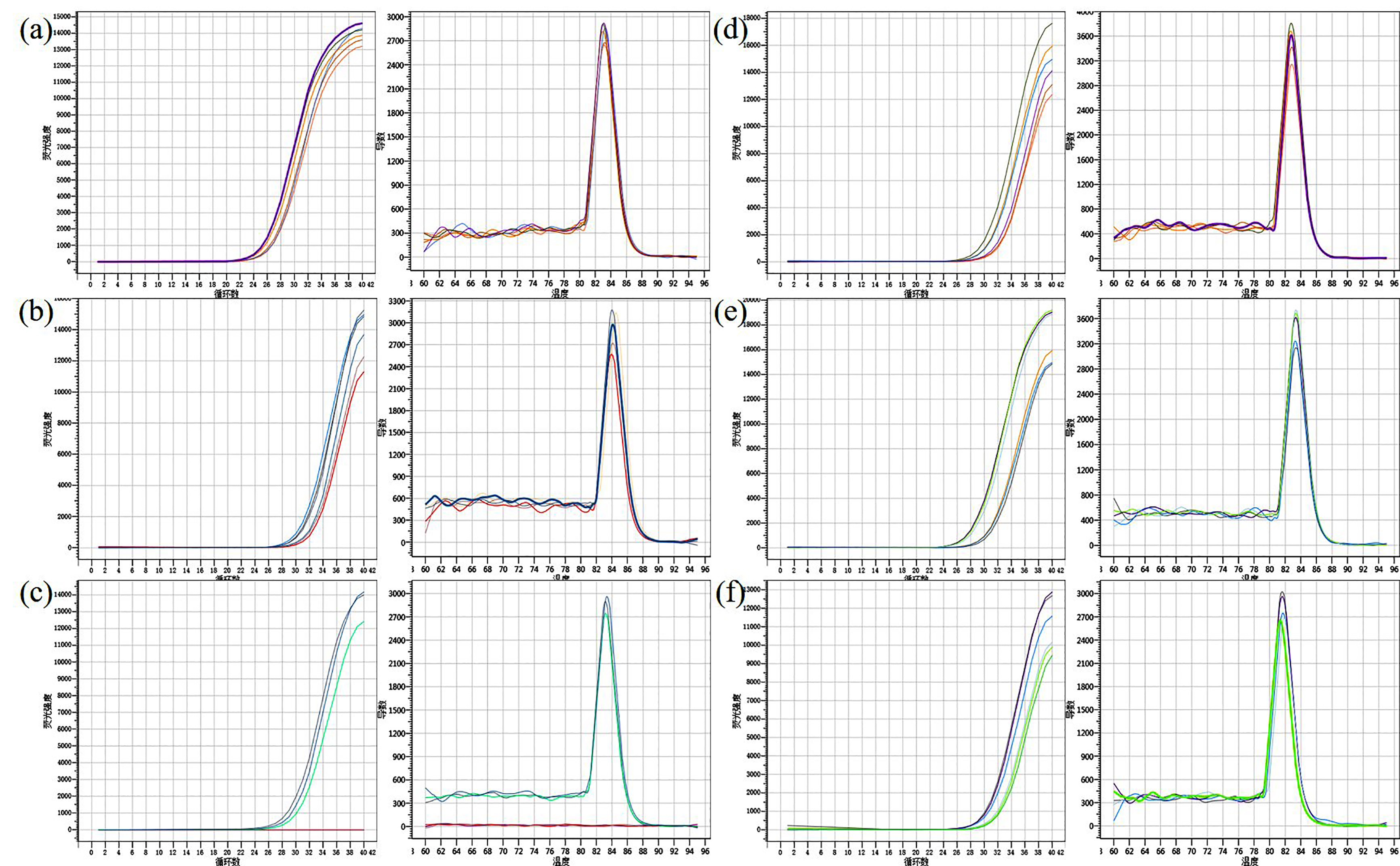

Supplement: Figure S2 — (A) qRT-PCR curve and Melt Curve for ACTIN7. (B) qRT-PCR curve and Melt Curve for Glyma.18G252800. (C) qRT-PCR curve and Melt Curve for Glyma.18G285800. (D) qRT-PCR curve and Melt Curve for Glyma.18G287400. (E) qRT-PCR curve and Melt Curve for Glyma.18G2298200. (f) qRT-PCR curve and Melt Curve for Glyma.02G211400. [file peerj-12-18252-s002.jpg]
